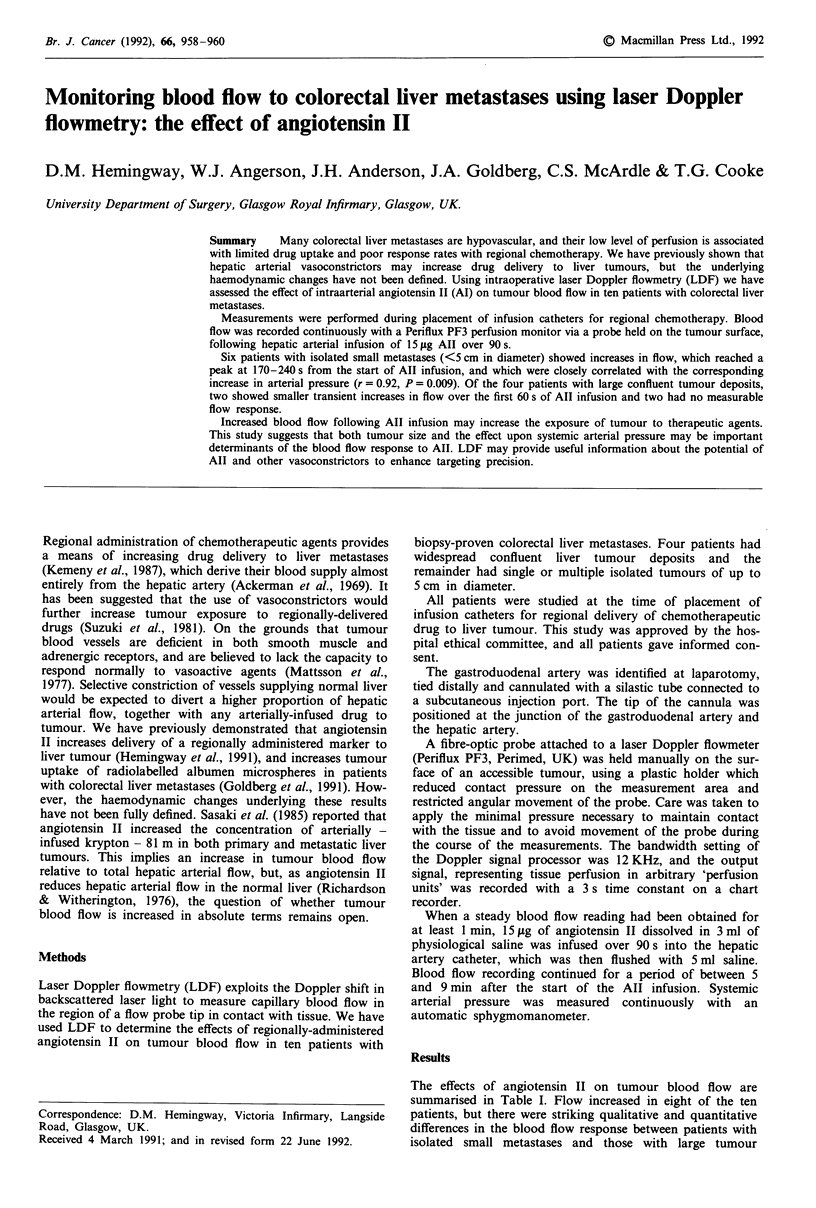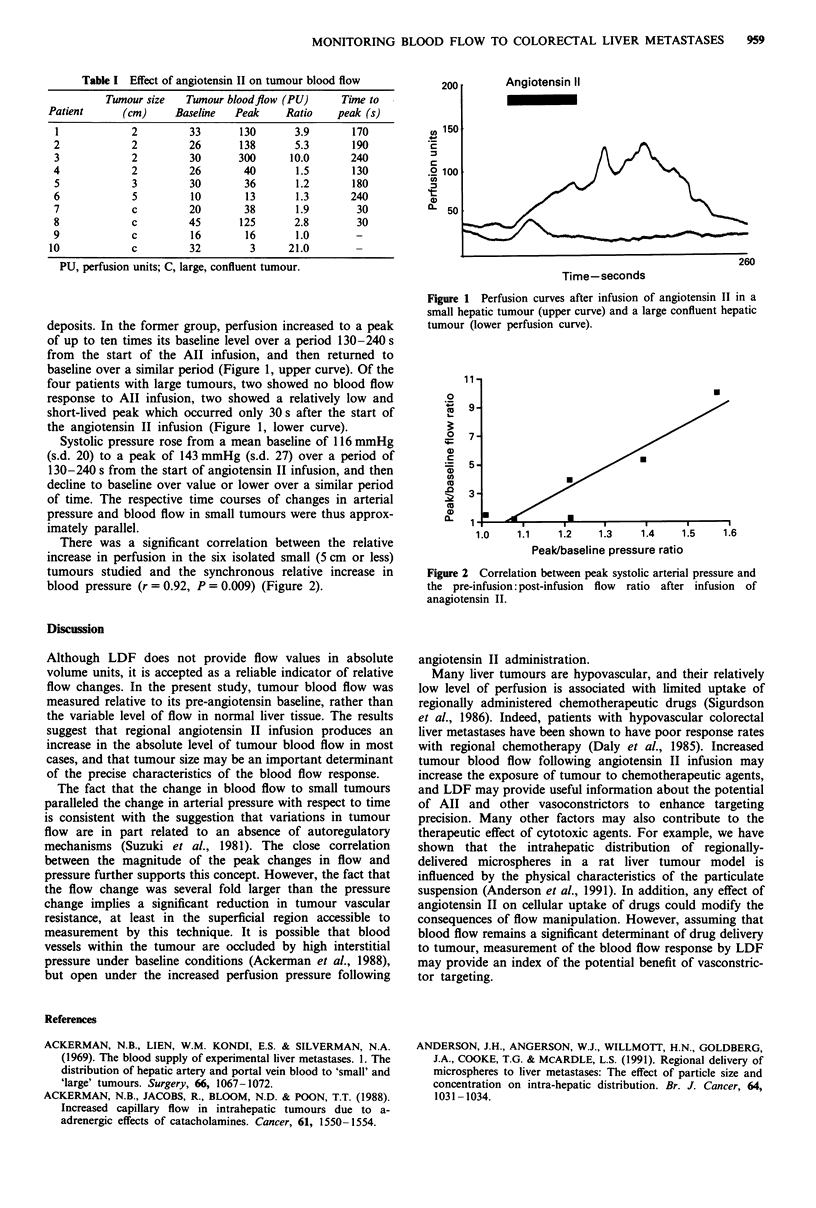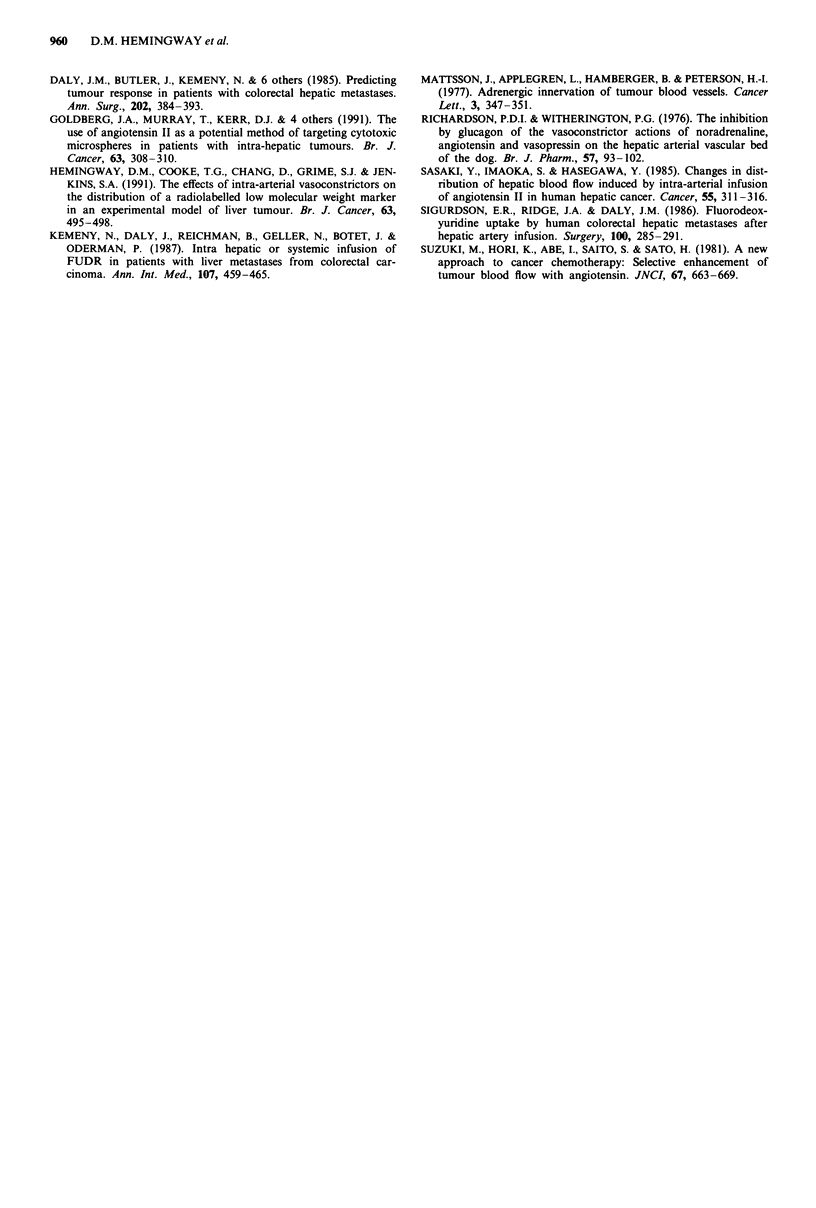# Monitoring blood flow to colorectal liver metastases using laser Doppler flowmetry: the effect of angiotensin II.

**DOI:** 10.1038/bjc.1992.392

**Published:** 1992-11

**Authors:** D. M. Hemingway, W. J. Angerson, J. H. Anderson, J. A. Goldberg, C. S. McArdle, T. G. Cooke

**Affiliations:** University Department of Surgery, Glasgow Royal Infirmary, UK.

## Abstract

Many colorectal liver metastases are hypovascular, and their low level of perfusion is associated with limited drug uptake and poor response rates with regional chemotherapy. We have previously shown that hepatic arterial vasoconstrictors may increase drug delivery to liver tumours, but the underlying haemodynamic changes have not been defined. Using intraoperative laser Doppler flowmetry (LDF) we have assessed the effect of intraarterial angiotensin II (AI) on tumour blood flow in ten patients with colorectal liver metastases. Measurements were performed during placement of infusion catheters for regional chemotherapy. Blood flow was recorded continuously with a Periflux PF3 perfusion monitor via a probe held on the tumour surface, following hepatic arterial infusion of 15 micrograms AII over 90 s. Six patients with isolated small metastases (< 5 cm in diameter) showed increases in flow, which reached a peak at 170-240 s from the start of AII infusion, and which were closely correlated with the corresponding increase in arterial pressure (r = 0.92, P = 0.009). Of the four patients with large confluent tumour deposits, two showed smaller transient increases in flow over the first 60 s of AII infusion and two had no measurable flow response. Increased blood flow following AII infusion may increase the exposure of tumour to therapeutic agents. This study suggests that both tumour size and the effect upon systemic arterial pressure may be important determinants of the blood flow response to AII. LDF may provide useful information about the potential of AII and other vasoconstrictors to enhance targeting precision.


Br. J. Cancer (1992), 66, 958–960 © Macmillan Press Ltd., 1992

### Monitoring blood flow to colorectal liver metastases using laser Doppler flowmetry: the effect of angiotensin II

D.M. Hemingway, W.J. Angerson, J.H. Anderson, J.A. Goldberg, C.S. McArdle & T.G. Cooke

*University Department of Surgery, Glasgow Royal Infirmary, Glasgow, UK.*

**Summary** Many colorectal liver metastases are hypovascular, and their low level of perfusion is associated with limited drug uptake and poor response rates with regional chemotherapy. We have previously shown that hepatic arterial vasoconstrictors may increase drug delivery to liver tumours, but the underlying haemodynamic changes have not been defined. Using intraoperative laser Doppler flowmetry (LDF) we have assessed the effect of intraarterial angiotensin II (AI) on tumour blood flow in ten patients with colorectal liver metastases.

Measurements were performed during placement of infusion catheters for regional chemotherapy. Blood flow was recorded continuously with a Periflux PF3 perfusion monitor via a probe held on the tumour surface, following hepatic arterial infusion of 15 μg AII over 90 s.

Six patients with isolated small metastases (<5 cm in diameter) showed increases in flow, which reached a peak at 170–240 s from the start of AII infusion, and which were closely correlated with the corresponding increase in arterial pressure ($r = 0.92$, $P = 0.009$). Of the four patients with large confluent tumour deposits, two showed smaller transient increases in flow over the first 60 s of AII infusion and two had no measurable flow response.

Increased blood flow following AII infusion may increase the exposure of tumour to therapeutic agents. This study suggests that both tumour size and the effect upon systemic arterial pressure may be important determinants of the blood flow response to AII. LDF may provide useful information about the potential of AII and other vasoconstrictors to enhance targeting precision.

Regional administration of chemotherapeutic agents provides a means of increasing drug delivery to liver metastases (Kemeny *et al.*, 1987), which derive their blood supply almost entirely from the hepatic artery (Ackerman *et al.*, 1969). It has been suggested that the use of vasoconstrictors would further increase tumour exposure to regionally-delivered drugs (Suzuki *et al.*, 1981). On the grounds that tumour blood vessels are deficient in both smooth muscle and adrenergic receptors, and are believed to lack the capacity to respond normally to vasoactive agents (Mattsson *et al.*, 1977). Selective constriction of vessels supplying normal liver would be expected to divert a higher proportion of hepatic arterial flow, together with any arterially-infused drug to tumour. We have previously demonstrated that angiotensin II increases delivery of a regionally administered marker to liver tumour (Hemingway *et al.*, 1991), and increases tumour uptake of radiolabelled albumen microspheres in patients with colorectal liver metastases (Goldberg *et al.*, 1991). However, the haemodynamic changes underlying these results have not been fully defined. Sasaki *et al.* (1985) reported that angiotensin II increased the concentration of arterially - infused krypton - 81 m in both primary and metastatic liver tumours. This implies an increase in tumour blood flow relative to total hepatic arterial flow, but, as angiotensin II reduces hepatic arterial flow in the normal liver (Richardson & Witherington, 1976), the question of whether tumour blood flow is increased in absolute terms remains open.

### Methods

Laser Doppler flowmetry (LDF) exploits the Doppler shift in backscattered laser light to measure capillary blood flow in the region of a flow probe tip in contact with tissue. We have used LDF to determine the effects of regionally-administered angiotensin II on tumour blood flow in ten patients with biopsy-proven colorectal liver metastases. Four patients had widespread confluent liver tumour deposits and the remainder had single or multiple isolated tumours of up to 5 cm in diameter.

All patients were studied at the time of placement of infusion catheters for regional delivery of chemotherapeutic drug to liver tumour. This study was approved by the hospital ethical committee, and all patients gave informed consent.

The gastroduodenal artery was identified at laparotomy, tied distally and cannulated with a silastic tube connected to a subcutaneous injection port. The tip of the cannula was positioned at the junction of the gastroduodenal artery and the hepatic artery.

A fibre-optic probe attached to a laser Doppler flowmeter (Periflux PF3, Perimed, UK) was held manually on the surface of an accessible tumour, using a plastic holder which reduced contact pressure on the measurement area and restricted angular movement of the probe. Care was taken to apply the minimal pressure necessary to maintain contact with the tissue and to avoid movement of the probe during the course of the measurements. The bandwidth setting of the Doppler signal processor was 12 KHz, and the output signal, representing tissue perfusion in arbitrary 'perfusion units' was recorded with a 3 s time constant on a chart recorder.

When a steady blood flow reading had been obtained for at least 1 min, 15 μg of angiotensin II dissolved in 3 ml of physiological saline was infused over 90 s into the hepatic artery catheter, which was then flushed with 5 ml saline. Blood flow recording continued for a period of between 5 and 9 min after the start of the AII infusion. Systemic arterial pressure was measured continuously with an automatic sphygmomanometer.

### Results

The effects of angiotensin II on tumour blood flow are summarised in Table I. Flow increased in eight of the ten patients, but there were striking qualitative and quantitative differences in the blood flow response between patients with isolated small metastases and those with large tumour

Correspondence: D.M. Hemingway, Victoria Infirmary, Langside Road, Glasgow, UK.
Received 4 March 1991; and in revised form 22 June 1992.

**Table I** Effect of angiotensin II on tumour blood flow

| *Patient* | *Tumour size (cm)* | *Tumour blood flow (PU) Baseline* | *Peak* | *Ratio* | *Time to peak (s)* |
|---|---|---|---|---|---|
| 1 | 2 | 33 | 130 | 3.9 | 170 |
| 2 | 2 | 26 | 138 | 5.3 | 190 |
| 3 | 2 | 30 | 300 | 10.0 | 240 |
| 4 | 2 | 26 | 40 | 1.5 | 130 |
| 5 | 3 | 30 | 36 | 1.2 | 180 |
| 6 | 5 | 10 | 13 | 1.3 | 240 |
| 7 | c | 20 | 38 | 1.9 | 30 |
| 8 | c | 45 | 125 | 2.8 | 30 |
| 9 | c | 16 | 16 | 1.0 | – |
| 10 | c | 32 | 3 | 21.0 | – |

PU, perfusion units; C, large, confluent tumour.

deposits. In the former group, perfusion increased to a peak of up to ten times its baseline level over a period 130–240 s from the start of the AII infusion, and then returned to baseline over a similar period (Figure 1, upper curve). Of the four patients with large tumours, two showed no blood flow response to AII infusion, two showed a relatively low and short-lived peak which occurred only 30 s after the start of the angiotensin II infusion (Figure 1, lower curve).

Systolic pressure rose from a mean baseline of 116 mmHg (s.d. 20) to a peak of 143 mmHg (s.d. 27) over a period of 130–240 s from the start of angiotensin II infusion, and then decline to baseline over value or lower over a similar period of time. The respective time courses of changes in arterial pressure and blood flow in small tumours were thus approximately parallel.

There was a significant correlation between the relative increase in perfusion in the six isolated small (5 cm or less) tumours studied and the synchronous relative increase in blood pressure ($r = 0.92$, $P = 0.009$) (Figure 2).

**Figure 1** Perfusion curves after infusion of angiotensin II in a small hepatic tumour (upper curve) and a large confluent hepatic tumour (lower perfusion curve).

**Figure 2** Correlation between peak systolic arterial pressure and the pre-infusion:post-infusion flow ratio after infusion of anagiotensin II.

### Discussion

Although LDF does not provide flow values in absolute volume units, it is accepted as a reliable indicator of relative flow changes. In the present study, tumour blood flow was measured relative to its pre-angiotensin baseline, rather than the variable level of flow in normal liver tissue. The results suggest that regional angiotensin II infusion produces an increase in the absolute level of tumour blood flow in most cases, and that tumour size may be an important determinant of the precise characteristics of the blood flow response.

The fact that the change in blood flow to small tumours paralleled the change in arterial pressure with respect to time is consistent with the suggestion that variations in tumour flow are in part related to an absence of autoregulatory mechanisms (Suzuki *et al.*, 1981). The close correlation between the magnitude of the peak changes in flow and pressure further supports this concept. However, the fact that the flow change was several fold larger than the pressure change implies a significant reduction in tumour vascular resistance, at least in the superficial region accessible to measurement by this technique. It is possible that blood vessels within the tumour are occluded by high interstitial pressure under baseline conditions (Ackerman *et al.*, 1988), but open under the increased perfusion pressure following angiotensin II administration.

Many liver tumours are hypovascular, and their relatively low level of perfusion is associated with limited uptake of regionally administered chemotherapeutic drugs (Sigurdson *et al.*, 1986). Indeed, patients with hypovascular colorectal liver metastases have been shown to have poor response rates with regional chemotherapy (Daly *et al.*, 1985). Increased tumour blood flow following angiotensin II infusion may increase the exposure of tumour to chemotherapeutic agents, and LDF may provide useful information about the potential of AII and other vasoconstrictors to enhance targeting precision. Many other factors may also contribute to the therapeutic effect of cytotoxic agents. For example, we have shown that the intrahepatic distribution of regionally-delivered microspheres in a rat liver tumour model is influenced by the physical characteristics of the particulate suspension (Anderson *et al.*, 1991). In addition, any effect of angiotensin II on cellular uptake of drugs could modify the consequences of flow manipulation. However, assuming that blood flow remains a significant determinant of drug delivery to tumour, measurement of the blood flow response by LDF may provide an index of the potential benefit of vasconstrictor targeting.

### References

ACKERMAN, N.B., LIEN, W.M. KONDI, E.S. & SILVERMAN, N.A. (1969). The blood supply of experimental liver metastases. 1. The distribution of hepatic artery and portal vein blood to 'small' and 'large' tumours. *Surgery*, **66**, 1067–1072.

ACKERMAN, N.B., JACOBS, R., BLOOM, N.D. & POON, T.T. (1988). Increased capillary flow in intrahepatic tumours due to a-adrenergic effects of catacholamines. *Cancer*, **61**, 1550–1554.

ANDERSON, J.H., ANGERSON, W.J., WILLMOTT, H.N., GOLDBERG, J.A., COOKE, T.G. & MCARDLE, L.S. (1991). Regional delivery of microspheres to liver metastases: The effect of particle size and concentration on intra-hepatic distribution. *Br. J. Cancer*, **64**, 1031–1034.

DALY, J.M., BUTLER, J., KEMENY, N. & 6 others (1985). Predicting tumour response in patients with colorectal hepatic metastases. *Ann. Surg.*, **202**, 384-393.

GOLDBERG, J.A., MURRAY, T., KERR, D.J. & 4 others (1991). The use of angiotensin II as a potential method of targeting cytotoxic microspheres in patients with intra-hepatic tumours. *Br. J. Cancer*, **63**, 308-310.

HEMINGWAY, D.M., COOKE, T.G., CHANG, D., GRIME, S.J. & JENKINS, S.A. (1991). The effects of intra-arterial vasoconstrictors on the distribution of a radiolabelled low molecular weight marker in an experimental model of liver tumour. *Br. J. Cancer*, **63**, 495-498.

KEMENY, N., DALY, J., REICHMAN, B., GELLER, N., BOTET, J. & ODERMAN, P. (1987). Intra hepatic or systemic infusion of FUDR in patients with liver metastases from colorectal carcinoma. *Ann. Int. Med.*, **107**, 459-465.

MATTSSON, J., APPLEGREN, L., HAMBERGER, B. & PETERSON, H.-I. (1977). Adrenergic innervation of tumour blood vessels. *Cancer Lett.*, **3**, 347-351.

RICHARDSON, P.D.I. & WITHERINGTON, P.G. (1976). The inhibition by glucagon of the vasoconstrictor actions of noradrenaline, angiotensin and vasopressin on the hepatic arterial vascular bed of the dog. *Br. J. Pharm.*, **57**, 93-102.

SASAKI, Y., IMAOKA, S. & HASEGAWA, Y. (1985). Changes in distribution of hepatic blood flow induced by intra-arterial infusion of angiotensin II in human hepatic cancer. *Cancer*, **55**, 311-316.

SIGURDSON, E.R., RIDGE, J.A. & DALY, J.M. (1986). Fluorodeoxyuridine uptake by human colorectal hepatic metastases after hepatic artery infusion. *Surgery*, **100**, 285-291.

SUZUKI, M., HORI, K., ABE, I., SAITO, S. & SATO, H. (1981). A new approach to cancer chemotherapy: Selective enhancement of tumour blood flow with angiotensin. *JNCI*, **67**, 663-669.